# Early outcomes of experience warm surgery in children undergoing complete repair of tetralogy of Fallot in developing countries

**DOI:** 10.1186/s12887-024-04976-9

**Published:** 2024-08-03

**Authors:** Alaa Mohamad Hussain, Mohammad Ali Younes

**Affiliations:** University Children’s Hospital, Damascus University, Damascus, Syrian Arab Republic

**Keywords:** Warm surgery, Hypothermia, Cardiopulmonary bypass, Tetralogy of Fallot, Early outcomes, Developing country

## Abstract

**Objectives:**

While significant evidence supports the benefits of normothermic cardiopulmonary bypass (NCPB) over hypothermic techniques, many institutions in developing countries, including ours, continue to employ hypothermic methods. This study aimed to assess the early postoperative outcomes of normothermic cardiopulmonary bypass (NCPB) for complete surgical repair via the Tetralogy of Fallot (TOF) within our national context.

**Methods:**

We conducted this study in the Pediatric Cardiac Intensive Care Unit (PCICU) at the University Children’s Hospital. One hundred patients who underwent complete TOF repair were enrolled and categorized into two groups: the normothermic group (*n* = 50, temperature 35–37 °C) and the moderate hypothermic group (*n* = 50, temperature 28–32 °C). We evaluated mortality, morbidity, and postoperative complications in the PCICU as outcome measures.

**Results:**

The demographic characteristics were similar between the two groups. However, the cardiopulmonary bypass (CPB) time and aortic cross-clamp (ACC) time were notably longer in the hypothermic group. The study recorded seven deaths, yielding an overall mortality rate of 7%. No significant differences were observed between the two groups concerning mortality, morbidity, or postoperative complications in the PCICU.

**Conclusions:**

Our findings suggest that normothermic procedures, while not demonstrably effective, are safe for pediatric cardiac surgery. Further research is warranted to substantiate and endorse the adoption of this technique.

## Introduction

The optimal temperature for cardiopulmonary bypass (CPB) during pediatric cardiac surgeries has historically been a subject of debate [1]. Traditionally, hypothermic perfusion was widely used based on the premise that lowering the body temperature would attenuate the inflammatory response triggered by CPB, offering protection to vital organs such as the brain, heart, and kidneys, particularly in scenarios involving unexpected technical failures during the CPB process [2]. However, recent studies have cast doubt on the long-held beliefs about hypothermic perfusion. Some research indicates that hypothermia may delay the inflammatory response rather than mitigate it [3–5]. This raises questions about the actual clinical benefits of using hypothermic perfusion. Additionally, the rarity of technical failures that could lead to circulatory arrest limits the justification of hypothermic perfusion.

In contrast, in the last two decades, there has been a surge in interest in normothermic cardiopulmonary bypass (NCPB), both in adult and pediatric cardiac surgeries [6–9].

The main potential concern toward using NCPB is the absence of a safe margin when an incident requiring circulatory arrest lasts more than a few minutes [9, 10]. Even though this disadvantage is known, NCPB is associated with lower oxidative stress, myocardial reperfusion injury, renal injury, and inflammatory response than is hypothermic cardiopulmonary bypass (HCPB) [5, 11]. Many studies have confirmed the benefits of warm perfusion on immediate outcomes through a low requirement for inotropic support, a short time to extubation, low lactate production, adequate urine output, minimal drainage from the chest drain, a short intensive care unit (ICU) stay, and a short length of hospital stay [7, 9, 12]. This has led several European centers to make the transition from hypothermic to normothermic procedures, with none opting to revert to the older method [13, 14].

Tetralogy of Fallot (TOF) is the most common form of cyanotic congenital heart disease in children. Despite advancements and the shift toward normothermic procedures in many developed countries, hypothermic perfusion remains the standard practice for the surgical repair of TOF in our national context. This study, therefore, aimed to fill this knowledge gap by comparing the outcomes of using normothermic and moderate hypothermic techniques during CPB. Specifically, we will focus on key metrics such as mortality rates, duration of patient intubation, length of stay in the pediatric cardiac intensive care unit (PCICU), postoperative hospital stays, and incidence of postoperative complications in children undergoing complete repair of TOF.

## Materials and methods

This comparison study was conducted in the PCICU at the University Children’s Hospital, Damascus. We evaluated 100 pediatric patients who underwent complete repair of TOF at the Cardiothoracic Unit of the Children’s Hospital from November 2018 to February 2023.

Ethical Considerations: Prior to the study, approval was secured from the hospital’s institutional ethics committee. Additionally, informed consent was obtained from the parents or guardians of the participating children.

### Study criteria

#### Inclusion criteria

Patients aged 13 years or younger who had undergone complete repair of TOF.

#### Exclusion criteria

Patients who required emergency surgery or surgeries conducted at temperatures ranging from 32 to 35 °C or below 28 °C or during periods of circulatory arrest.

Excluding patients within the 32–35 °C range was essential to clearly differentiate between the two main approaches and to avoid potential confusion from overlapping results.

Temperature Definitions and Grouping: Ambiguities in the definitions of normothermic perfusion and hypothermia led us to define normothermia as ≥ 35 °C, as referenced in [15]. Consequently, patients were categorized into two groups:

1. NCPB Group (50 patients): Surgeries were performed at temperatures between 35 and 37 °C.
2. Patients in the moderate HCPB Group (50 patients): Surgeries were conducted at temperatures between 28 and 32 °C.

Temperature monitoring was achieved using nasopharyngeal and rectal probes.

Surgical Procedure: All surgeries during the study period were performed by a consistent surgeon employing analogous techniques. Standard CPB conditions with intermittent warm blood cardioplegia were maintained. For every patient, ventricular septal defect (VSD) closure via a transatrial approach was performed. Patients with valvular-PS underwent either commissurotomy or valvulotomy. Complete infundibular muscle resection was conducted, followed by enlargement of the outflow pathway using a bovine pericardial patch. If the right ventricular outflow tract obstruction persisted after pulmonary valvotomy and muscle resection, a transannular patch (TAP) was applied using a bovine pericardial patch.

### Data collection

Medical records were meticulously reviewed to extract pertinent clinical variables, encompassing demographic information such as age, body weight, body surface area, incidence of cyanotic spells, and sex. Parents were admitted to the cardiac intensive care unit postoperatively for informed consent and verification of their past medical history. Data acquisition was facilitated by the Drager–Infinity Delta kappa, which enables event recording. Preoperative data collection commenced at the time of surgery, during which the patient was a daily activity. The key metrics recorded included CPB and aortic cross-clamp (ACC) times, the lowest CPB temperature during surgery, and the necessity for a TAP. Postoperative data included intubation duration, intensive care unit (ICU) stay duration, total hospital stay, mortality incidence, and postoperative complications such as acute renal injury, neurological complications, arrhythmias, pulmonary complications, sepsis, bleeding, high inotropic support dosage, low cardiac output syndrome, and heart block. Continuous ECG monitoring was facilitated by Drager monitors, with standard 12-lead ECGs taken preoperatively and upon ICU admission.

### Definitions

High inotropic agent doses in the ICU were defined as doses exceeding 0.5 mcg/kg/min for epinephrine and norepinephrine and 10 mcg/kg/min for dopamine and dobutamine.

Pulmonary complications were defined as the presence of pneumonia, pleural effusion, pulmonary edema, pulmonary hemorrhage, pneumothorax, or pneumomediastinum.

Neurological Complications Assessment: This assessment was confined to clinical examinations, excluding electroencephalogram (EEG) or magnetic resonance imaging (MRI) evaluations. Medical personnel documented any transient or permanent neurological deficits, seizures, irritability, or aberrant behavior.

Acute Renal Injury: Either an absolute creatinine value increase of more than 0.3 mg/dL within 48 h or a urinary output of less than 0.5 ml/kg/hour for 6 h.

Postoperative low cardiac output syndrome: The presence of two or more of the following criteria:

1. The serum lactate concentration was more than 3 mmol/L.
2. Urinary output was less than 0.5 ml/kg/hour.
3. The difference between the skin temperature and central temperature was more than 5 degrees.
4. A drop in blood pressure below the 5% line, a weak pulse, or a prolonged capillary refill time.
5. Tachycardia above normal rates for age.

### Outcomes

This study focused on assessing the influence of cardiopulmonary bypass (CPB) temperature on key clinical metrics, including mortality rates, duration of intensive care unit (ICU) stay, overall hospital stay, and intubation time. These metrics were chosen to determine the overall effectiveness and safety of using normothermic versus hypothermic CPB techniques in pediatric patients undergoing complete repair of Tetralogy of Fallot (TOF).

### Statistical analysis

Descriptive statistics were calculated as frequency counts and percentages for categorical variables and medians with interquartile ranges (IQRs) for continuous variables. We used the Mann‒Whitney test to compare continuous variables, and the chi-square test was used to compare categorical variables between the NCPB and the HCPB groups. We used univariate logistic regression to assess the associations between mortality and postoperative complications and between mortality and the CPB temperature. We also used univariate Cox regression to assess the associations between the length of hospital stay and CPB temperature. *P* < 0.05 was considered to indicate statistical significance. All analyses were performed using SPSS 26 software (IBM SPSS Statistics for Windows, Version 26.0. Armonk, NY: IBM Corp.).

## Results

### Baseline characteristics

During this study, 100 patients who underwent complete repair of TOF were included, and they were divided into two groups: normothermic (50 patients) and moderate hypothermic (50 patients). Our findings indicate that both groups were demographically homogeneous, with no statistically significant differences in variables such as age, body weight, body mass index, sex distribution, or incidence of cyanotic spells, as delineated in Table 1. In addition, the median age at the time of operation was 2 years, with an IQR of 1–3 years. Statistical analysis revealed no significant difference between the two groups (*p* value = 0.76).

**Table 1:** Comparison of the baseline characteristics between the two groups

variable	Total (*n* = 100)	Normothermia	Hypothermia	*P* value
		**(n = 50) 50%**	**(n = 50) 50%**	
Age, median (IQR) years	2 (1–3)	2 (1–3)	2 (0.9–3.3)	0.76
Sex, Male (%)	63%	56%	70%	0.14
Weight, median (IQR) kg	10 (8.5–13)	10 (8.8–13)	10 (8.4–12)	0.8
BSA, median (IQR) m^2^	0.48 (0.42–0.56)	0.48 (0.43–0.57)	0.47 (0.41–0.54)	0.63
Cyanotic spells (%)	55%	54%	56%	0.84
Right aortic arch (%)	18%	16%	20%	-

BSA: body surface area, IQR: Interquartile range

### Intraoperative data

To assess the impact of temperature on surgical duration, we compared the CPB and ACC times between the hypothermic and normothermic groups. Our findings revealed that the hypothermic group had a longer CPB duration by an average of 10 min, with a median time of 115 min (IQR 77.5–97.75), in contrast to the normothermic group’s median of 104 min (IQR 89–118.5). This difference was statistically significant, as indicated by a *p* value of 0.008.

Similarly, the ACC time was notably different between the two groups. The median ACC time in the hypothermic group was 88 min (IQR 5.9–25.3), approximately 10 min longer than that in the normothermic group, which had a median of 76 min (IQR 66.5–92). This difference was also statistically significant, with a *p* value of 0.009.

In summary, both the CPB and ACC durations were significantly longer in the hypothermic group than in the normothermic group. These differences are further detailed in Table 2.

**Table 2:** Comparison of the intraoperative data between the two groups

variable	Total (*n* = 100)	Normothermia	Hypothermia	*P* value
		**(n = 50) 50%**	**(n = 50) 50%**	
ACC time, median (IQR) min	84 (70–94)	76 (66.5–92)	88 (77.5–98)	0.009
CPB time, median (IQR) min	109 (95–122)	104 (89-118.5)	115 (103–126)	0.008
TAP (%)	7%	4%	10%	0.24

ACC: aortic cross-clamp, CPB: cardiopulmonary bypass, TAP: transannular patch, IQR: Interquartile range

### Outcomes

To evaluate the impact of temperature modulation on patient outcomes, we analyzed various metrics including mortality, hospitalization duration, mechanical ventilation time, and duration of ICU stay, as well as postoperative complications between the hypothermic and normothermic groups. Our findings indicated that out of the total participants, seven died, for an overall mortality rate of 7%. The specific causes of mortality in our study were as follows: heart failure led to the death of 4 patients, septic shock was responsible for the deaths of 2 patients, and pulmonary hemorrhage led to the death of 1 patient.

After a greater delay, the hypothermic group exhibited a greater mortality rate—5 deaths (10%)—than did the normothermic group (4%). However, this difference was not statistically significant (*P* value = 0.25).

In terms of hospitalization duration, the hypothermic group had a slightly shorter median hospital stay (6 days; IQR 5–9) than did the normothermic group (median 7 days; IQR 5.75-10). However, this difference did not reach statistical significance (*p* value = 0.75). Similarly, both groups had comparable median mechanical ventilation times (20 h) and ICU stay durations (3 days), but neither difference was statistically significant (*P* values of 0.84 and 0.75, respectively).

When assessing the requirement for high doses of inotropic support, 34% of the patients in the normothermic group had this condition compared to 44% of those in the hypothermic group. This difference, however, was not statistically significant (*p* value = 0.3).

Finally, regarding other postoperative complications in the ICU, such as acute renal injury, neurological complications, pulmonary complications, and bleeding, no significant differences were observed between the two groups (with *p* values of 0.56, 0.2, 0.59, and 0.65, respectively).

In summary, while there were observable differences in outcomes between the hypothermic and normothermic groups, they did not reach statistical significance. A detailed comparison of the postoperative data for both groups can be found in Table 3.

**Table 3:** Comparison of the postoperative data between the two groups

variable	Total (*n* = 100)	Normothermia	Hypothermia	Exp B (95% CI)	*P* value
		**(n = 50) 50%**	**(n = 50) 50%**		
Mortality (%)	7%	4%	10%	0.37 (0.07–2.03)	0.25
Length of hospital stay, median (IQR) days	6.5 (5-9.75)	7 (5.75-10)	6 (5-9.25)	1.07 (0.71–1.61)	0.75
Length of PCICU stay, median (IQR) days	3 (2-4.75)	3 (2–5)	3 (2-4.25)	-	0.75
Intubation time, median (IQR) hours	20 (8–37)	20 (8-43.25)	20 (7-32.5)	-	0.84
Acute renal injury (%)	3%	2%	4%	2 (0.18–23.26)	0.56
Pulmonary complications (%)	26%	26%	26%	1 (0.4–2.44)	0.59
Neurological complications (%)	5%	2%	8%	4.2 (0.46–39.54)	0.2
Bleeding (%)	5%	4%	6%	1.5 (0.24–9.59)	0.65
Sepsis (%)	29%	34%	24%	0.61 (0.25–1.47)	0.27
Heart Block (%)	1%	2%	0%	-	0.99
Arrhythmias (%)	20%	16%	24%	1.66 (0.61–4.49)	0.32
LCOS (%)	26%	20%	32%	1.9 (0.75–4.69)	0.17
High Dose of Inotropic Support (%)	39%	34%	44%	1.52 (0.68–3.42)	0.3

Lcos: low cardiac output syndrome, PCICU: pediatric cardiac intensive care unit, IQR: Interquartile range

## Discussion

NCPB in pediatric cardiac surgery has garnered increased attention due to its demonstrated safety and efficacy and has become a prevalent practice in numerous European cardiac centers. Despite its popularity elsewhere, this technique remains underutilized in our country, prompting us to conduct this study. We aimed to evaluate the early outcomes of performing normothermic CPB in children who underwent complete surgical repair of TOF, with a particular focus on its safety and feasibility in our local context.

In this study, descriptive statistics showed that the median age of the children was 2 years (IQR 1–3), which was greater than the age mentioned in the study of Mercer-Rosa, L. et al. conducted at Philadelphia Hospital in 2018 [16], where the median age was 3.6 months (interquartile range 2.1, 5.2). We can explain the older ages in this study by the delay in diagnosis and the delay in surgery for patients due to the large number of patients relative to the center’s ability to reach admission and due to the insufficient availability of centers specializing in pediatric heart surgery in other regions of our country.

The overall mortality rate in the current study was 7%, which was higher than that reported in other studies (less than 2%) [16–18]. This increase can be linked to the challenging economic and health conditions in our country stemming from ongoing conflict, which leads to shortages of essential medical supplies and equipment, particularly in operating rooms and ICUs. The mortality rate was greater in the HCPB group (10%) than in the normothermic group (4%), although these differences did not reach statistical significance (*P* = 0.25). These findings align with previous studies that also reported no significant difference in mortality rates between the two temperature groups [7, 11]. Our results revealed no significant differences between the hypothermic and normothermic groups in terms of hospital stay, ICU stay, or mechanical ventilation time (*P* values of 0.75, 0.75, and 0.84, respectively). These findings are consistent with those reported by Xiong et al. and Caputo et al. [7, 19], although they contrast with the findings of other studies [8]. Postoperative complications in the ICU were generally more prevalent in the HCPB group, but this difference was not statistically significant, in agreement with prior research [7, 19].

A critical observation from our study was the longer CPB duration in the HCPB group, which was attributed to the additional time required for cooling at the start and rewarming at the end of CPB. The NCPB group also exhibited a shorter ACC duration, likely because the aortic cross-clamp was used solely for intracardiac defect repairs, while right ventricular outflow tract reconstruction was performed on a beating heart. In contrast, the HCPB group underwent the entire procedure with the aortic cross-clamp in place, with heart reperfusion occurring only after the required rewarming period. These findings are in line with those of several previous studies [9, 11], though two other studies reported no significant differences in CPB or ACC times between the two groups [12, 20].

Notably, the difference between the two groups concerning the CPB and ACC times was small, approximately 10 min, which likely explains the lack of impact on surgical repair outcomes observed in our study.

### Study limitations

Despite these insights, our study is not without limitations. The relatively small sample size and the challenging conditions of the health system in our country introduced variables that could have influenced the outcomes, and the unstable availability of laboratory materials prevented a comprehensive laboratory variable analysis.

We acknowledge that utilizing the area under the curve (AUC) method to analyze temperature and flow rate data could provide valuable insights into the detailed physiological dynamics during surgery. However, due to limited resources and technical capabilities at the time of designing this study, we were unable to implement this approach. Future studies should incorporate AUC analysis to provide a more nuanced understanding of temperature management and its impact on patient outcomes. This will help in offering a comprehensive evaluation of the different CPB techniques.

### Study strengths

This study has several strengths, including the consistency of surgical experience and technique, the fact that all the operations were performed by the same surgeon, and the homogeneity of the patient sample, as all the participants had classical TOF disease. Additionally, the elective nature of the surgeries eliminated the potential influence of surgical repair complexity on the results.

## Conclusion

This study found that although the duration of aortic cross-clamp (ACC) and cardiopulmonary bypass (CPB) was shorter in the normothermic cardiopulmonary bypass (NCPB) group compared to the hypothermic cardiopulmonary bypass (HCPB) group, the early postoperative outcomes did not significantly differ between the two groups of patients undergoing surgical repair of Tetralogy of Fallot (TOF). This suggests that employing a warm surgical approach, even if not more effective, is safe in children with TOF. However, to further substantiate the applicability and benefits of this technique, it is imperative to expand the research to include other congenital heart diseases. Future studies in this direction could provide a more comprehensive understanding and validation of the technique’s potential benefits across a broader spectrum of pediatric cardiac conditions.

By focusing on enhancing surgical training, resource allocation, policy advocacy, and early diagnosis, we aim to reduce mortality rates and improve overall outcomes in pediatric cardiac surgery. We believe these measures will significantly contribute to the advancement of pediatric cardiac care in our region, ensuring our patients receive the best possible care under challenging circumstances.

## Data Availability

The datasets used and analysed during the current study available from the corresponding author on reasonable request.

## Abbreviations

NCPB: Normothermic cardiopulmonary bypass
TOF: Tetralogy of Fallot
PCICU: Pediatric Cardiac Intensive Care Unit
CPB: Cardiopulmonary bypass
ACC: Aortic cross-clamp
HCPB: Hypothermic cardiopulmonary bypass
ICU: Intensive care unit
VSD: Ventricular septal defect
TAP: Transannular patch
EEG: Electroencephalogram
MRI: Magnetic resonance imaging
IQR: Interquartile ranges
